# GC Gene Polymorphisms and Vitamin D-Binding Protein Levels Are Related to the Risk of Generalized Aggressive Periodontitis

**DOI:** 10.1155/2016/5141089

**Published:** 2016-11-28

**Authors:** Wenli Song, Xian'e Wang, Yu Tian, Xin Zhang, Ruifang Lu, Huanxin Meng

**Affiliations:** ^1^Department of Periodontology, Peking University School and Hospital of Stomatology, Beijing, China; ^2^Department of General Dentistry, First Dental Center, Peking University School and Hospital of Stomatology, Beijing, China; ^3^Department of Stomatology, Peking Union Medical College Hospital, Chinese Academy of Medical Science, Beijing, China

## Abstract

*Objective*. To explore whether GC (group-specific component) rs17467825, rs4588, and rs7041 polymorphisms are associated with generalized aggressive periodontitis.* Methods*. This case-control study recruited 372 patients with generalized aggressive periodontitis (group AgP) and 133 periodontal healthy subjects (group HP). GC rs17467825, rs4588, and rs7041 genotypes and plasmatic vitamin D-binding protein (DBP) were measured. Analysis of single SNP and multiple SNPs was performed and relevance between plasmatic DBP and haplotypes was analyzed.* Results*. GC rs17467825 GG genotype was statistically associated with lower risk for generalized aggressive periodontitis under the recessive model (OR = 0.52, 95% CI: 0.30–0.92, *p* = 0.028). GC rs17467825 and rs4588 had strong linkage disequilibrium with *r* ^2^ ≥ 0.8 and *D*′ ≥ 0.8. Haplotype (GC rs17467825, rs4588) GC was associated with the less risk for generalized aggressive periodontitis (OR = 0.29, 95% CI: 0.09–0.96, *p* = 0.043). In group AgP, individuals with combined genotype (GC rs17467825, rs4588) AG+CA had significantly lower plasmatic DBP level than those with the other two combined genotypes (AG+CA versus AA+CC *p* = 0.007; AG+CA versus GG+AA *p* = 0.026).* Conclusions*. GC rs17467825 genotype GG and haplotype (GC rs17467825, rs4588) GC are associated with generalized aggressive periodontitis. The association may be acquired through regulating DBP levels. The functions of GC gene and DBP in inflammatory disease need to be further studied.

## 1. Introduction

Periodontitis is primarily a chronic infectious disease. With its incidence as high as 47%–68.97%, it is the main cause of tooth loss in adults [1–3]. It not only affects oral health, function, and aesthetics, but also has strong association with endocrine disturbance (e.g., diabetes mellitus, obesity, and osteoporosis) and cardiovascular disease [4–8]. Aggressive periodontitis, as an important branch of periodontitis, is characterized by early onset, severe periodontal destruction and familial aggregation. In view of the fact that the incidence rates of cardiovascular diseases and endocrine diseases are increasing among young people, exploring the etiology of aggressive periodontitis and sequentially controlling periodontal inflammation may contribute to controlling these diseases and maintaining homeostasis [9–12].

Vitamin D-binding protein (DBP), known as gc-globulin (group-specific component, GC), is a member of albumin gene family, together with serum albumin and alpha-fetoprotein. DBP plays an important role in the transport and metabolism of vitamin D. It also participants in actin scavenging, monocyte response to C5-derived peptides enhancing, macrophage activation, and so on [13–15]. Previous studies of our group showed that DBP level was significantly elevated in the plasma of patients with generalized aggressive periodontitis than that in healthy controls. It indicated that DBP was closely associated with generalized aggressive periodontitis.

Recent studies of aggressive periodontitis pay more attention to single nucleotide polymorphisms (SNPs) because the disease has significant familial aggregation, which indicates that genetic predisposition may be an important etiological factor of aggressive periodontitis [16, 17]. DBP is encoded by the GC gene, which is located on chromosome 4 (4q11–q13) and coded by 1690 nucleotides. The rs17467825 is located in the 3′-UTR region of the GC gene. This SNP could be in strong linkage disequilibrium (LD) with causal functional variants affecting the mRNA stability of DBP [18]. The two most common polymorphisms rs7041 (c.1296T>G encoding D432E) and rs4588 (c.1307C>A encoding T436K) are located in exon 11. Powe et al. found racial variation and GC SNPs (rs4588 and rs7041) explained nearly 80% of DBP concentration difference in black and white Americans [19]. These three sites were reported to be associated with bone mineral density, obesity, and 25-hydroxyvitamin D concentration, which were important factors for periodontitis [4–8]. Xiong et al. genotyped 1873 subjects from 405 white nuclear families across 20 genes and found GC rs17467825 was associated with spine bone mineral density [20]. Individuals with rs17467825 GG were confirmed to have the lowest 25-hydroxyvitamin D concentration in Arab Asians [4]. Suaini et al. found that rs17467825 minor allele was associated with increased odds of vitamin D insufficiency [21]. The same group also found this site showed strongest association with percentage of fat mass. Ezura et al. reported that D432E (rs7041) in conjunction with IVS1+827C>T showed the strongest relationship with bone mineral density (*p* = 0.005) [22]. Almesri et al. recruited 406 subjects and found rs7041 GG and rs4588 CC were associated with high body mass index in females (*p* = 0.003, *p* = 0.034) [23]. It was also reported that rs7041 GG was associated with the lower risk of Parkinson's Disease [24]. So the aims of this study are to explore whether GC gene polymorphisms are associated with generalized aggressive periodontitis and to investigate the relationship between GC genotype and DBP level.

## 2. Material and Methods

### 2.1. Study Population

From July 2001 to December 2013, a total of 372 unrelated Chinese individuals diagnosed as generalized aggressive periodontitis and 133 periodontal healthy subjects were recruited in this case-control study. They were all from the Clinic of Periodontology Department, Peking University School and Hospital of Stomatology. The diagnosis of generalized aggressive periodontitis was based on the 1999 International Classification of Periodontal Diseases and Condition.

At baseline, the inclusion criteria of generalized aggressive periodontitis group (group AgP) were (1) being under 35 years of age at the time that the disease was diagnosed and (2) having at least six teeth left (at least three of which were not incisors or first molars) with probing depth (PD) ≥ 5 mm and clinical attachment loss (CAL) ≥ 3 mm. Individuals with PD ≤ 3 mm or without obvious attachment loss were defined as periodontal healthy controls (group HP). Exclusion criteria of all subjects were (1) history of periodontal therapy, history of orthodontic therapy, or antimicrobial therapy within 6 months and (2) systemic disease (e.g., diabetes mellitus, cardiovascular disease, and rheumatoid arthritis) or being pregnant or under medication known to affect the periodontium.

PD and CAL measurements were taken at six sites (i.e., mesiobuccal, buccal, distobuccal, distolingual, lingual, and mesiolingual) for each tooth, excluding third molars. William's periodontal probe was used in the measurements. The mean of PD and AL for each person was analyzed.

The study was approved by Ethic Committee of Peking University Health Science Center and all participants had signed consent forms.

### 2.2. DNA Collection and Genotyping

A total 5 mL of fasting blood was taken from all participants through venipuncture between 8:00 am and 10:00 am and injected into a vacuum tube with EDTA. Plasma was isolated and stored at −80°C while WBC was used for DNA extraction. DNA was extracted from all samples using a blood DNA mini kit (Watson Biotechnologies, Inc., Shanghai, China), following the manufacturer's instructions. In 2009, our group selected 122 SNPs in 38 genes to study the association between SNPs and aggressive periodontitis. These SNPs were reported in the literatures or GenBank to be associated with immunoinflammatory responses, lipid metabolism, glucose metabolism and bone metabolism, hormone metabolism, and periodontal tissue growth. At that time, three SNPs (GC rs17467825, rs4588, and rs7041) in GC gene were reported. These three SNPs were genotyped by Shanghai Benegene Biotechnology Co., Ltd. using the MassARRAY time of flight mass spectrometry (MALDI-TOF) platform from Sequenom®. And primer sequences of the three sites were as follows:

rs17467825
  Primer 1: 5′-ACGTTGGATGCAATATTTCTGTCAGCGATTC-3′
  Primer 2: 5′-ACGTTGGATGTTCCAGCACACTCTAAACAC-3′
rs4588
  Primer 1: 5′-ACGTTGGATGGCTTGTTAACCAGCTTTGCC-3′
  Primer 2: 5′-ACGTTGGATGGTTTTTCAGACTGGCAGAGC-3′
rs7041
  Primer 1: 5′-ACGTTGGATGGTTTTTCAGACTGGCAGAGC-3′
  Primer 2: 5′-ACGTTGGATGGCTTGTTAACCAGCTTTGCC-3′

### 2.3. Measurement of Plasmatic DBP Levels

Plasmatic DBP level was measured with ELISA method using plasma samples mentioned above. The commercially available ELISA kit was from BioSource Systems, Invitrogen, Grand Island, NY, USA. The assay was performed according to the manufacturer's protocols. The lower limit of plasmatic DBP detection was 7.81 *μ*g/mL. With the limit of expenses and time, 145 participants' plasma samples were measured, 54 in group HP and 91 in group AgP, respectively.

### 2.4. Statistics Analysis

All data of continuous variables were tested for normal distribution by Kolmogorov-Smirnov test. The differences of participant characteristics in different groups and different genotypes were explored using chi-square tests for categorical variables and *t*-tests for continuous variables. Plasmatic DBP levels in different combined genotypes were compared by one-way analysis of variance while comparison between two groups adopted least significance difference test. Distribution differences between all participants and participants with DBP tested were compared by nonparameter testing. These analyses were carried out using software SPSS (version 19.0). Analysis of single SNPs (multiple inheritance models, Hardy-Weinberg equilibrium) and multiple SNPs (haplotype frequency estimation, analysis of association between haplotypes and disease condition) was performed using web tool SNPStats (http://bioinfo.iconcologia.net/snpstats/start.htm). Linkage disequilibrium (LD) analysis was carried out by software HaploView, version 4.2. *p* < 0.05 was considered statistically significant.

## 3. Results

### 3.1. Basic Characteristics of the Study Population

Characteristics of all participants in the two groups were given in the Table 1. There were no significant differences in age and gender between the two groups. PD and AL in group AgP are significantly higher than those in group HP (4.85 ± 1.06 versus 1.76 ± 0.46 mm, *p* < 0.01; 4.45 ± 1.52 versus 0 mm, *p* < 0.01). Plasmatic DBP of 145 participants were analyzed, 54 in group HP and 91 in group AgP, respectively. The results were also displayed in Table 1. Individuals in group AgP had significantly higher plasmatic DBP level than those in group HP (238.89 ± 49.89 versus 110.96 ± 21.47 *μ*g/mL, *p* < 0.01).

### 3.2. Genotype Distribution

Genotype detecting rates of GC rs17467825, rs4588, and rs7041 were, respectively, 97.2%, 99.0%, and 98.2%. GC rs17467825, rs4588, and rs7041 polymorphisms met Hardy Weinberg equilibrium (HWE). Genotype frequency distributions were displayed in Table 2. Adjusted by age and gender, rs17467825 GG genotype was associated with lower risk for generalized aggressive periodontitis and the difference was statistically significant in recessive model (OR = 0.52, 95% CI: 0.30–0.92, *p* = 0.028). The rs4588 and rs7041 were not statistically associated with generalized aggressive periodontitis in all inheritance models. We used Akaike's information criterion (AIC) and Bayesian information criterion (BIC) to determine the best-fit model for each SNP. For GC rs17467825 site, the recessive model was much more powerful than the others.

### 3.3. Linkage Disequilibrium (LD) Analysis

**Figure 1:**
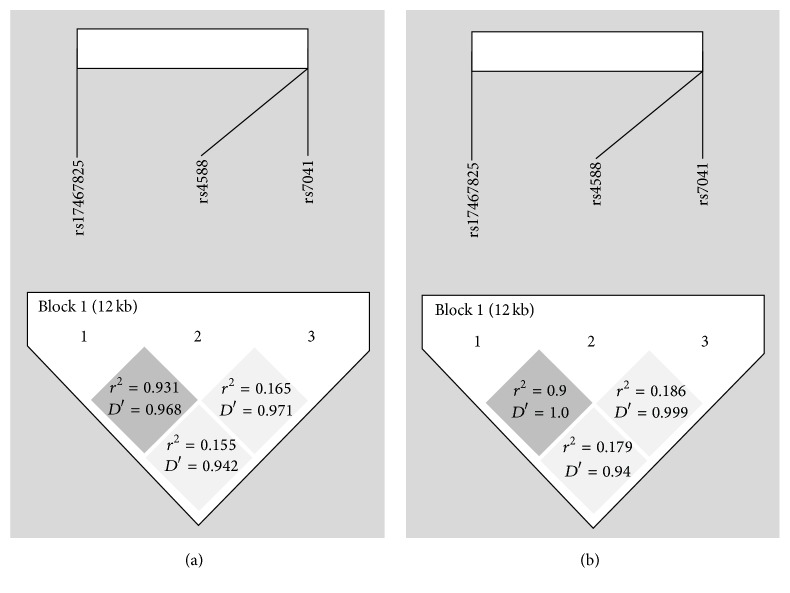
Linkage disequilibrium analysis of DBP SNPs. (a) represents LD analysis in group AgP and (b) represents the result in group HP. The values in the diamonds represent the *r* ^2^ and *D*′ values.

LD analysis was an effective way to identify the combined function of the genes and LD was calculated for these three SNPs in the study.  Figure 1 showed that the disequilibrium coefficient was greater between rs174567825 and rs4588 in both groups (group AgP *D*′ = 0.968, LOD = 126.6, *r* ^2^ = 0.931; group HP *D*′ = 1.0, LOD = 47.82, *r* ^2^ = 0.9), while the disequilibrium coefficients, especially *r* ^2^ values, were much lower for the pairs of rs4588-rs7041 (group AgP: *D*′ = 0.971, LOD = 17.26, *r* ^2^ = 0.165; group HP: *D*′ = 0.999, LOD = 8.82, *r* ^2^ = 0.186) and rs174567825-rs7041 (group AgP: *D*′ = 0.942, LOD = 15.6, *r* ^2^ = 0.155; group HP: *D*′ = 0.94, LOD = 8.02, *r* ^2^ = 0.179).

### 3.4. Haplotype Analysis of GC rs17467825 and rs4588 Polymorphisms and Their Association with Generalized Aggressive Periodontitis

Haplotype analysis of rs17467825 and rs4588 polymorphisms was carried out because we found that rs17467825 and rs4588 had strong linkage disequilibrium (Table 3). Three haplotypes (GC rs17467825 and rs4588) AC, GA, and GC accounted for 99.16% in group AgP and 100% in group HP. Haplotype AC with the most frequent alleles was defined as the reference. Furthermore, the haplotype GC was associated with the lower risk for generalized aggressive periodontitis (OR = 0.29, 95% CI: 0.09–0.96, *p* = 0.043).

### 3.5. Combined Genotype Distributions and Their Association with Plasmatic DBP Level

**Figure 2:**
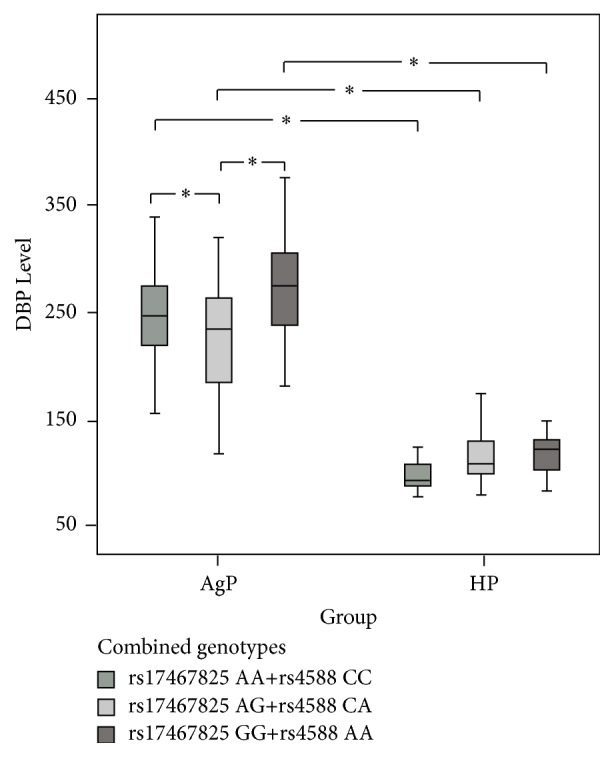
Plasmatic DBP level comparison between individuals with different combined genotypes. AgP: aggressive periodontitis; HP: healthy participant; DBP: vitamin D-binding protein; ^*∗*^ *p* < 0.05: statistical significance.

Combined genotype frequencies were displayed in Table 4. Combined genotypes with genotype frequency ≤0.01 were not displayed. Three combined genotypes (GC rs17467825, rs4588) AA+CC, AG+CA, and GG+AA accounted for 96.74% in all participants and 93.79% in participants with DBP measured. There were no statistical differences in their distributions (group AgP *p* = 0.254; group HP *p* = 0.332). Plasmatic DBP levels of individuals with different combined genotypes were compared in Figure 2. With same combined genotypes, individuals in group AgP had statistically higher DBP levels than those in group HP (AA+CC 246.79 ± 44.16 versus 101.89 ± 19.18 *μ*g/mL, *p* < 0.01; AG+CA 226.42 ± 49.04 versus 116.15 ± 21.82 *μ*g/mL, *p* < 0.01; GG+AA 275.19 ± 64.73 versus 117.48 ± 21.5 *μ*g/mL, *p* < 0.01). In group AgP, individuals with combined genotype AG+CA had statistically lower plasmatic DBP level than those with the other two combined genotypes (AG+CA versus AA+CC *p* = 0.007; AG+CA versus GG+AA *p* = 0.026). Although individuals with combined genotype GG+AA seemed to have higher plasmatic DBP level than those with combined genotype AA+CC, there was no statistical difference (AA+CC versus GG+AA *p* = 0.104). No significant differences were found in group HP (AG+CA versus AA+CC *p* = 0.256; AG+CA versus GG+AA *p* = 0.934; AA+CC versus GG+AA *p* = 0.330).

## 4. Discussion

Infectious periodontal tissues acting as focus have powerful and multiple influences on the occurrence and severity of systemic diseases and abnormal status. Recent studies have reported that periodontitis is associated with diabetes mellitus, cardiovascular disease, obesity, osteoporosis, respiratory diseases, and pregnancy complications [5–8, 25–28]. In those patients combined with severe periodontitis, periodontal treatment may help control these diseases [9–12]. Uncontrolled periodontitis affects patients' quality of life and overburdens the entire healthcare system to a certain degree. Thus it is time to pay more attention to the periodontal health.

The incidence rate of aggressive periodontitis is very low. Three surveys in local areas of China reported that the incidence rate of aggressive periodontitis is 0.12%–0.47% [29]. The incidences in different areas and different races are different, which may be influenced by ethnic differences of gene polymorphisms [30]. Lots of studies have investigated the importance of genetic polymorphisms (e.g., MCP-1, S100A8, IL-1*α*, IL-1*β*, and N-formyl peptide receptor) in aggressive periodontitis [31–34]. To the best knowledge of the authors, there are only two studies that have investigated the association between GC gene and periodontitis. A family study recruiting 19 unrelated families showed the restriction fragment length polymorphism (RFLP) at the GC locus did not have linkage with early onset periodontitis in 1993 [35]. The other study of a five-generation family from southern Maryland discovered that GC locus had close linkage with that determining the autosomal-dominant form of juvenile periodontitis in 1986 [36]. This study is the first attempt to associate GC gene SNPs with generalized aggressive periodontitis. We find that rs17467825 GG genotype is statistically related to less risk for generalized aggressive periodontitis in the recessive model while rs4588 and rs7041 genotype distributions are not confirmed to correlate with generalized aggressive periodontitis. In a case-control study, Bakke et al. found that GC rs17467825 was associated with chronic obstructive pulmonary disease (COPD) and the risk allele was A. The result was consistent with ours. It seems that rs17467825 allele G is more likely to be associated with lower risk for morbid state [37].

Aggressive periodontitis is a polygenic disorder and genetic predisposition of this disease results from interactions among lots of gene loci. Thus haplotypes may be more effective to predict diseases. A haplotype is a group of genes that tend to travel through generations as a block and its existence is based on LD [38]. It has been observed that GC rs174567825 and rs4588 are linked together with *r* ^2^ ≥ 0.8 and *D*′ ≥ 0.8. As the disequilibrium coefficients for the pairwise rs174567825-rs7041 and rs4588-rs7041 are *D*′ ≥ 0.8 and *r* ^2^ ≤ 0.8, weak evidences can be provided for their genetic linkage. Through the haplotype analysis, we find that haplotype (GC rs17467825, rs4588) GC is associated with the lower risk for generalized aggressive periodontitis with OR = 0.29. This OR value is much smaller than that of rs17467825 site (OR = 0.52) resulting from single SNP analysis. Compared with rs17467825 site alone, the haplotypes of rs174567825 and rs4588 improve the predictability of aggressive periodontitis. To our best knowledge, there are no correlative literatures about genetic linkage of these two sites. However, GC rs4588 and rs7041 are reported to be linked together in several studies [39–43]. Genetic distribution and transmission vary from race to race. There is one study carried out in Chinese population. Li et al. recruited 1,443 unrelated employees and retired workers from a factory in Dali (25°N), Yunnan Province, China [39]. After LD analysis of rs4588 and rs7041, the disequilibrium coefficient (*r* ^2^ = 0.16) was really close to our result. In addition to the disequilibrium coefficient, minor allele frequencies (rs4588 0.30; rs7041 0.28) were also similar to ours (rs4588 0.32; rs7041 0.27).

Although there is no statistical difference, individuals with combined genotype (GC rs17467825, rs4588) GG+AA seem to have higher plasmatic DBP level than those with combined genotype AA+CC. Medlej-Hashim et al. collected blood samples from 128 university students and found participants with genotypes rs4588 AA had higher concentrations of DBP in a young Lebanese population [44]. The result was similar to ours. Previous studies of our group showed that DBP level was significantly elevated in the plasma of patients with generalized aggressive periodontitis [45]. Trujillo et al. established different animal models of alveolitis and found that neutrophil recruitment was significantly reduced in the lungs of DBP(−/−) mice compared with their wild-type DBP(+/+). After histological examination, statistically less injury was found in DBP(−/−) mouse lungs than that in wild-type mice [46]. GalNAc-modified Gc protein (DBP-MAF) converted from DBP is a potent macrophage activating factor. Schneider et al. injected newborn rats of osteopetrosis with DBP-MAF every 4 days. Increased number of osteoclasts was found in these rats treated by DBP-MAF and the majority of them exhibited normal structure [47, 48]. In these studies, high DBP level seems to be associated with strong inflammatory response and osteoclastogenesis. In group AgP, individuals with combined genotype (GC rs17467825, rs4588) AG+CA, which are more likely to have haplotype (GC rs17467825, rs4588) GC, have statistically lower plasmatic DBP level than those with the other two combined genotypes. That may explain why haplotype (GC rs17467825, rs4588) GC was associated with the lower risk for aggressive periodontitis.

## Figures and Tables

**Table 1:** Basic characteristics of the study population.

	Total	Group HP	Group AgP	*p* ^*∗*^
Sample size	505	133	372	—
Age^a^	27.83 ± 5.79	28.77 ± 7.04	27.50 ± 5.24	0.058
Male/female	205/300	53/80	152/220	0.839
*PD (mm)* ^a^	*4.06 ± 1.65*	*1.76 ± 0.46*	*4.85 ± 1.06*	*<0.01*
*AL (mm)* ^a^	*3.32 ± 0.11*	*0*	*4.45 ± 1.52*	*<0.01*
DBP (*μ*g/mL)^a^	191.25 ± 74.69	110.96 ± 21.47	238.89 ± 49.89	*<0.01*

AgP: aggressive periodontitis; HP: healthy participant; DBP: vitamin D-binding protein; *PD: probing depth; AL: clinical attachment loss*. ^a^Data were presented as mean ± SD. ^*∗*^Analyzed by Student's *t*-test for continuous variable and by Pearson's chi-square test for categorical variable.

**Table 2:** Genotype distributions of GC rs17467825, rs4588, and rs7041 polymorphisms in the inheritance models.

Model	Genotypes	Group HP *N* (%)	Group AgP *N* (%)	Adjusted OR (95% CI)	Adjusted *p*	AIC	BIC
*rs17467825*							
Codominant	A/A	62 (48.4%)	170 (46.8%)	1	0.040	564.8	581.6
	A/G	43 (33.6%)	156 (43%)	1.33 (0.85–2.08)			
	G/G	23 (18%)	37 (10.2%)	0.59 (0.33–1.08)			
Dominant	A/A	62 (48.4%)	170 (46.8%)	1	0.730	569.1	581.7
	A/G-G/G	66 (51.6%)	193 (53.2%)	1.07 (0.72–1.61)			
Recessive	A/A-A/G	105 (82%)	326 (89.8%)	*1*	*0.028* ^*∗*^	564.4	577
	G/G	23 (18%)	37 (10.2%)	*0.52 (0.30–0.92)*			
Overdominant	A/A-G/G	85 (66.4%)	207 (57%)	1	0.059	565.7	578.3
	A/G	43 (33.6%)	156 (43%)	1.49 (0.98–2.28)			
Log-additive	—	—	—	0.88 (0.66–1.18)	0.410	568.6	581.1

*rs4588*							
Codominant	C/C	65 (49.2%)	168 (45.6%)	1.00	0.190	581.9	598.7
	C/A	48 (36.4%)	163 (44.3%)	1.32 (0.86–2.03)			
	A/A	19 (14.4%)	37 (10.1%)	0.76 (0.40–1.41)			
Dominant	C/C	65 (49.2%)	168 (45.6%)	1.00	0.470	582.7	595.3
	C/A-A/A	67 (50.8%)	200 (54.4%)	1.16 (0.78–1.73)			
Recessive	C/C-C/A	113 (85.6%)	331 (90%)	1.00	0.190	581.4	594.1
	A/A	19 (14.4%)	37 (10.1%)	0.67 (0.37–1.20)			
Overdominant	C/C-A/A	84 (63.6%)	205 (55.7%)	1.00	0.110	580.6	593.3
	C/A	48 (36.4%)	163 (44.3%)	1.39 (0.93–2.10)			
Log-additive	—	—	—	0.99 (0.73–1.32)	0.920	583.2	595.8

*rs7041*							
Codominant	T/T	68 (51.9%)	190 (52%)	1.00	0.970	580.6	597.5
	G/T	54 (41.2%)	152 (41.6%)	1.01 (0.66–1.53)			
	G/G	9 (6.9%)	23 (6.3%)	0.92 (0.40–2.08)			
Dominant	T/T	68 (51.9%)	190 (52%)	1.00	0.980	578.7	591.3
	G/T-G/G	63 (48.1%)	175 (48%)	0.99 (0.67–1.48)			
Recessive	T/T-G/T	122 (93.1%)	342 (93.7%)	1.00	0.820	578.6	591.3
	G/G	9 (6.9%)	23 (6.3%)	0.91 (0.41–2.03)			
Overdominant	T/T-G/G	77 (58.8%)	213 (58.4%)	1.00	0.930	578.7	591.3
	G/T	54 (41.2%)	152 (41.6%)	1.02 (0.68–1.53)			
Log-additive	—	—	—	0.98 (0.71–1.36)	0.910	578.7	591.3

AgP: aggressive periodontitis; HP: healthy participant; OR: odds ratio; 95% CI: 95% confidence interval; AIC: Akaike's information criterion; BIC: Bayesian information criterion; HWE: Hardy Weinberg equilibrium; adjusted *p*: adjusted by age and gender; ^*∗*^ *p* < 0.05: statistical significance.

**Table 3:** Haplotype analysis of GC rs17467825, rs4588, and rs7041 polymorphisms and their association with generalized aggressive periodontitis.

	Haplotypes		Haplotype frequency			OR (95% CI)	Adjusted *p*
	rs17467825	rs4588	Total	Group HP	Group AgP		
1	A	C	0.6664	0.6509	0.6719	1	—
2	G	A	0.3161	0.3258	0.3127	0.95 (0.70–1.28)	0.74
3	G	C	0.0112	0.0234	0.0069	*0.29 (0.09–0.96)*	*0.043* ^*∗*^
4	A	A	0.0062	0.0084	NA	—	—

AgP: aggressive periodontitis; HP: healthy participant; OR: odds ratio; 95% CI: 95% confidence interval; NA: not analyzed; adjusted *p*: adjusted by age and gender; ^*∗*^ *p* < 0.05: statistical significance.

**Table 4:** Combined genotype distributions in different groups ^a^.

rs17467825	rs4588	All participants			Participants with DBP measured		
		Total	HP	AgP	Total	HP	AgP
AA	CC	228 (46.53%)	62 (48.44%)	166 (45.86%)	67 (46.21%)	21 (38.89%)	46 (50.55%)
AG	CA	193 (39.39%)	41 (32.03%)	152 (41.99%)	54 (37.24%)	20 (37.04%)	34 (37.36%)
GG	AA	53 (10.82%)	19 (14.84%)	34 (9.39%)	15 (10.34%)	9 (16.67%)	6 (6.60%)
*∗*	*∗*	16 (3.26%)	6 (4.69%)	10 (2.76%)	9 (6.21%)	4 (7.40%)	5 (5.49%)

AgP: aggressive periodontitis; HP: healthy participant; DBP: vitamin D-binding protein; ^*∗*^combined genotypes with genotype frequency ≤0.01 were not displayed, including (GC rs17467825, rs4588) AA+CA, AA+AA, AG+CC, AG+AA, GG+CC, and GG+CA; ^a^data were presented as *N* (%).

## References

[1] Zhang Q., Li Z., Wang C. (2014). Prevalence and predictors for periodontitis among adults in China, 2010. *Global Health Action*.

[2] Li Z., Zhou J., Hu X., Yu Z., Ma L., Lian W. (2013). Oral health status and its correlation with oral health knowledge among middle-aged people in Dongxiang, Bonan, and Yugur. *Hua Xi Kou Qiang Yi Xue Za Zhi*.

[3] Eke P. I., Dye B. A., Wei L., Thornton-Evans G. O., Genco R. J. (2012). Prevalence of periodontitis in adults in the United States: 2009 and 2010. *Journal of Dental Research*.

[4] Elkum N., Alkayal F., Noronha F. (2014). Vitamin D insufficiency in Arabs and South Asians positively associates with polymorphisms in GC and CYP2R1 genes. *PLoS ONE*.

[5] Stewart R., West M. (2016). Increasing evidence for an association between periodontitis and cardiovascular disease. *Circulation*.

[6] Moura-Grec P. G., Marsicano J. A., Carvalho C. A. P., Sales-Peres S. H. D. C. (2014). Obesity and periodontitis: systematic review and meta-analysis. *Ciencia e Saude Coletiva*.

[7] Straka M., Straka-Trapezanlidis M., Deglovic J. (2015). Periodontitis and osteoporosis. *Neuroendocrinology Letters*.

[8] Malik S., Fu L., Juras D. J. (2013). Common variants of the vitamin D binding protein gene and adverse health outcomes. *Critical Reviews in Clinical Laboratory Sciences*.

[9] Mammen J., Vadakkekuttical R. J., George J. M., Kaziyarakath J. A., Radhakrishnan C. (2016). Effect of non-surgical periodontal therapy on insulin resistance in patients with type II diabetes mellitus and chronic periodontitis, as assessed by C-peptide and the Homeostasis Assessment Index. *Journal of Investigative and Clinical Dentistry*.

[10] Botero J. E., Rodríguez C., Agudelo-Suarez A. A. (2016). Periodontal treatment and glycaemic control in patients with diabetes and periodontitis: an umbrella review. *Australian Dental Journal*.

[11] Torumtay G., Kirzioğlu F. Y., Öztürk Tonguç M., Kale B., Calapoğlu M., Orhan H. (2016). Effects of periodontal treatment on inflammation and oxidative stress markers in patients with metabolic syndrome. *Journal of Periodontal Research*.

[12] Carallo C., De Franceschi M. S., Tripolino C. (2015). Periodontal treatment elevates carotid wall shear stress in the medium term. *Medicine*.

[13] Speeckaert M., Huang G., Delanghe J. R., Taes Y. E. C. (2006). Biological and clinical aspects of the vitamin D binding protein (Gc-globulin) and its polymorphism. *Clinica Chimica Acta*.

[14] Piquette C. A., Robinson-Hill R., Webster R. O. (1994). Human monocyte chemotaxis to complement-derived chemotaxins is enhanced by Gc-globulin. *Journal of Leukocyte Biology*.

[15] White P., Cooke N. (2000). The multifunctional properties and characteristics of vitamin D-binding protein. *Trends in Endocrinology and Metabolism*.

[16] Rapp G. E., Pineda-Trujillo N., McQuillin A., Tonetti M. (2010). Genetic power of a Brazilian three-generation family with generalized aggressive periodontitis. *Brazilian Dental Journal*.

[17] Vieira A. R., Albandar J. M. (2014). Role of genetic factors in the pathogenesis of aggressive periodontitis. *Periodontology 2000*.

[18] Fang Y., Van Meurs J. B. J., D'Alesio A. (2005). Promoter and 3′-untranslated-region haplotypes in the vitamin D receptor gene predispose to osteoporotic fracture: The Rotterdam Study. *American Journal of Human Genetics*.

[19] Powe C. E., Evans M. K., Wenger J. (2013). Vitamin D-binding protein and vitamin D status of black Americans and white Americans. *The New England Journal of Medicine*.

[20] Xiong D.-H., Shen H., Zhao L.-J. (2006). Robust and comprehensive analysis of 20 osteoporosis candidate genes by very high-density single-nucleotide polymorphism screen among 405 white nuclear families identified significant association and gene-gene interaction. *Journal of Bone and Mineral Research*.

[21] Suaini N. H. A., Koplin J. J., Ellis J. A. (2014). Environmental and genetic determinants of vitamin D insufficiency in 12-month-old infants. *Journal of Steroid Biochemistry and Molecular Biology*.

[22] Ezura Y., Nakajima T., Kajita M. (2003). Association of molecular variants, haplotypes, and linkage disequilibrium within the human vitamin D-binding protein (DBP) gene with postmenopausal bone mineral density. *Journal of Bone and Mineral Research*.

[23] Almesri N., Das N. S., Ali M. E., Gumaa K., Giha H. A. (2016). Independent associations of polymorphisms in vitamin D binding protein (GC) and vitamin D receptor (VDR) genes with obesity and plasma ^25^OHD_3_ levels demonstrate sex dimorphism. *Applied Physiology, Nutrition, and Metabolism*.

[24] Gezen-Ak D., Alaylioglu M., Genc G. (2016). GC and VDR SNPs and vitamin D levels in Parkinson's disease: the relevance to clinical features. *Neuromolecular Medicine*.

[25] Kumar M., Mishra L., Mohanty R., Nayak R. (2014). Diabetes and gum disease: the diabolic duo. *Diabetes and Metabolic Syndrome: Clinical Research and Reviews*.

[26] Meng H.-X. (2007). Association between periodontitis and diabetes mellitus. *Beijing Da Xue Xue Bao*.

[27] Bansal M., Khatri M., Taneja V. (2013). Potential role of periodontal infection in respiratory diseases—a review. *Journal of Medicine and Life*.

[28] Papapanou P. N. (2015). Systemic effects of periodontitis: lessons learned from research on atherosclerotic vascular disease and adverse pregnancy outcomes. *International Dental Journal*.

[29] Meng H. (2014). *Clinical Periodontology*.

[30] Zhang J., Sun X., Xiao L., Xie C., Xuan D., Luo G. (2011). Gene polymorphisms and periodontitis. *Periodontology 2000*.

[31] Zhu X. L., Meng H. X., Zhang L. (2012). Association analysis between the -2518MCP-1(A/G) polymorphism and generalized aggressive periodontitis in a Chinese population. *Journal of Periodontal Research*.

[32] Sun X., Meng H., Shi D. (2011). Analysis of plasma calprotectin and polymorphisms of S100A8 in patients with aggressive periodontitis. *Journal of Periodontal Research*.

[33] Li Q. Y., Zhao H. S., Meng H. X. (2004). Association analysis between interleukin-1 family polymorphisms and generalized aggressive periodontitis in a Chinese population. *Journal of Periodontology*.

[34] Zhu X.-L., Meng H.-X., Zhang L. (2009). Association of SNPs in N-formylpeptide receptor gene with susceptibility of aggressive periodontitis. *Beijing Da Xue Xue Bao*.

[35] Hart T. C., Marazita M. L., McCanna K. M., Schenkein H. A., Diehl S. R. (1993). Reevaluation of the chromosome 4q candidate region for early onset periodontitis. *Human Genetics*.

[36] Boughman J. A., Halloran S. L., Roulston D. (1986). An autosomal-dominant form of juvenile periodontitis: its localization to chromosome 4 and linkage to dentinogenesis imperfecta and Gc. *Journal of Craniofacial Genetics and Developmental Biology*.

[37] Bakke P. S., Zhu G., Gulsvik A. (2011). Candidate genes for COPD in two large data sets. *European Respiratory Journal*.

[38] Crawford D. C., Nickerson D. A. (2005). Definition and clinical importance of haplotypes. *Annual Review of Medicine*.

[39] Li L.-H., Yin X.-Y., Wu X.-H. (2014). Serum 25(OH)D and vitamin D status in relation to VDR, GC and CYP2R1 variants in Chinese. *Endocrine Journal*.

[40] Lafi Z. M., Irshaid Y. M., El-Khateeb M., Ajlouni K. M., Hyassat D. (2015). Association of rs7041 and rs4588 polymorphisms of the vitamin D binding protein and the rs10741657 polymorphism of CYP2R1 with vitamin D status among jordanian patients. *Genetic Testing and Molecular Biomarkers*.

[41] Mateos-Muñoz B., García-Martín E., Torrejón M. J. (2016). GC gene polymorphism and unbound serum retinol-binding protein 4 are related to the risk of insulin resistance in patients with chronic hepatitis C. *Medicine*.

[42] Madden K., Feldman H. A., Chun R. F. (2015). Critically ill children have low Vitamin D-binding protein, influencing bioavailability of Vitamin D. *Annals of the American Thoracic Society*.

[43] Zhang Z., He J.-W., Fu W.-Z., Zhang C.-Q., Zhang Z.-L. (2013). An analysis of the association between the vitamin D pathway and serum 25-hydroxyvitamin D levels in a healthy Chinese population. *Journal of Bone and Mineral Research*.

[44] Medlej-Hashim M., Jounblat R., Hamade A. (2015). Hypovitaminosis D in a young lebanese population: effect of GC gene polymorphisms on vitamin D and vitamin D binding protein levels. *Annals of Human Genetics*.

[45] Zhang X., Meng H., Sun X. (2013). Elevation of vitamin D-binding protein levels in the plasma of patients with generalized aggressive periodontitis. *Journal of Periodontal Research*.

[46] Trujillo G., Habiel D. M., Ge L., Ramadass M., Cooke N. E., Kew R. R. (2013). Neutrophil recruitment to the lung in both C5a- and CXCL1-induced alveolitis is impaired in vitamin D-binding protein-deficient mice. *The Journal of Immunology*.

[47] Nagasawa H., Uto Y., Sasaki H. (2005). Gc protein (vitamin D-binding protein): Gc genotyping and GcMAF precursor activity. *Anticancer Research*.

[48] Schneider G. B., Benis K. A., Flay N. W., Ireland R. A., Popoff S. N. (1995). Effects of vitamin D binding protein-macrophage activating factor (DBP-MAF) infusion on bone resorption in two osteopetrotic mutations. *Bone*.

